# Single-Site Local-Density Potentials for the Mesoscopic Representation of Water Based on the SAFT-VR Mie Equation of State

**DOI:** 10.1021/acs.jpcb.4c06454

**Published:** 2025-01-30

**Authors:** James P. D. O’Connor, Ian P. Stott, Andrew J. Masters, Carlos Avendaño

**Affiliations:** † Department of Chemical Engineering, School of Engineering, The University of Manchester, Oxford Road, Manchester M13 9PL, U.K.; ‡ Unilever Research & Development Port Sunlight, Quarry Road East, Bebington CH63 3JW, U.K.

## Abstract

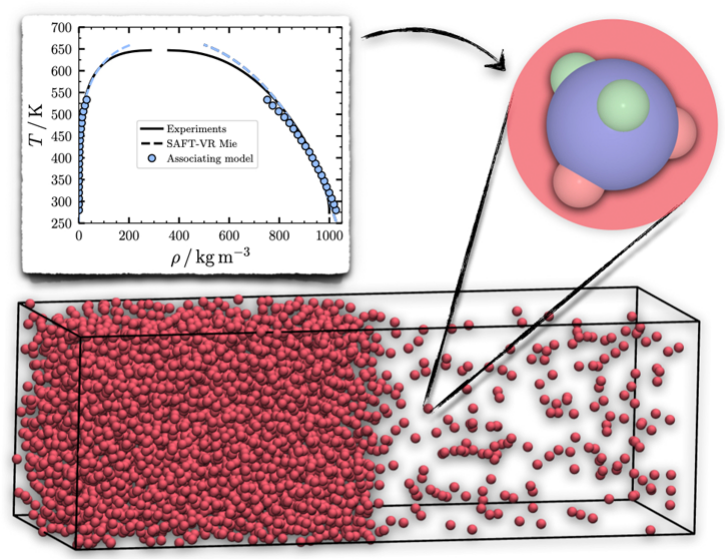

In this article, we present three mesoscopic models for water. All three models make use of local density-dependent interaction potentials, as employed within the Pagonabarraga-Frenkel framework [PagonabarragaI.; FrenkelD.J. Chem. Phys.2001, 115, 5015–5026]. The forms of these three interaction potentials are based on the free energy function of the SAFT-VR Mie equation of state (EoS) [LafitteT.J. Chem. Phys.2013, 139, 15450424160524 10.1063/1.4819786]. Two of these models represent the water–water interaction as a spherically symmetric Mie interaction with temperature-dependent parameters, while the third model works with a temperature-independent Mie potential and explicitly models the effect of hydrogen bonding using an association term. All three models provide good predictions of the vapor–liquid equilibrium of water over a wide temperature range. They also give accurate predictions of the isothermal compressibility for both sub- and supercritical conditions. To model the interfacial tension of the vapor–liquid interface with our mesoscale simulations, we added a square-gradient term to our potential energy function. We show that the addition of this term has a minimal effect on the bulk properties of water. However, by parametrizing the coefficient of this term as a function of temperature, all three models again provide excellent predictions of water’s interfacial tension over a wide temperature range. Of the three models, our preference is for the model that includes an association term, as this model can operate successfully over a wider range of conditions.

## 1 Introduction

Water plays a central role in life and is possibly, as the most abundant liquid on Earth, the most common solvent used in scientific and industrial applications. It is widely used in the healthcare industry as a solvent for many soft-matter formulations such as shampoos and other health-care products.^1^ Despite the importance of water in our lives, modeling its properties remains a challenge. While good, atomistic models exist to describe the properties of water,^2,^ these cannot be used to simulate aqueous soft matter systems, as these would be prohibitively costly given the length and time scales one needs to probe for such systems. This situation has prompted the development of several coarse-grained (CG) water models with different levels of complexity.^4^

In coarse-graining, atoms are lumped together into large beads with a certain degree of coarse-graining, that is, the number of atoms in each CG bead. This degree of coarse graining has a profound impact on the accuracy of the description of the properties of a system. In general, the higher the degree of coarse graining, the lower the accuracy and transferability of the model. For the representation of water in soft matter, typical CG models represent multiple molecules as a single-site model. Some common examples of single-site models using simple pairwise interactions are the MARTINI force field,^5,6^ the force field of Shinoda et al.^7−9^ and the SAFT-γ-Mie force field.^10−15^ Despite the widespread use of these models, the short-range repulsive forces prevent the use of either the large time steps in molecular dynamics or the large particle displacements in Monte Carlo simulations, which are required to describe soft matter phenomena that operate on long length and time scales.

In order to access longer time and length scales, techniques such as dissipative particle dynamics (DPD) have been developed.^16,17^ These make use of a soft, bounded pair potential which allows for large time steps. When these soft potentials are combined with a suitable thermostat, the methodology can be used to study self-assembling systems.^18,19^ However, this method does have limitations, particularly in relation to the accurate description of thermodynamic properties. Thus, one would not expect standard DPD water models, for example, to yield realistic values of the pressure, entropy, and enthalpy. The standard DPD potential results in a quadratic equation of state (EoS) which does not allow for vapor–liquid equilibria,^16,20,21^ which, in turn, prevents the study of free interfaces. We note, however, a recent model, known as m-DPD, which can model vapor–liquid equilibrium.^22^

A way to improve this situation is to make use of local-density potentials (LDPs). Here, for a given configuration of particles, one calculates an instantaneous local density associated with each particle in the system.^20,21,23,24^ These local densities are then used to calculate the total potential energy of the system, using a potential energy that is a function of these local densities. The internal energy described can have many purposes, from refining a traditional pairwise coarse-grained potential,^24−28^ to producing a soft, DPD-like potential with more desirable thermodynamic properties.^18,20,21,23,29,30^ As a result, these models allow the formation of vapor–liquid interfaces.^20,21^ In the context of DPD, this approach is commonly known as many-body DPD and has been used to describe many systems in soft matter, including a recent application to surfactants.^18^ One particular LDP methodology of significant importance is the one proposed by Pagonabarraga and Frenkel (PF),^20,23^ in which the LDP is designed to produce a fluid with the approximate thermophysical properties of an EoS of choice, allowing one to choose *a priori* the thermodynamics of their system from the top down. In the following, this potential will be denoted as LDP-PF and is the focus of this study. We also note that the gen-DPD approach is equivalent to the LDP-PF in the limit that the simulated particles have no internal degrees of freedom.^200^

The LDP-PF methodology requires a careful choice of an EoS that describes the thermodynamic properties of the system. There are many equations of state that can be adopted to represent the thermodynamics of the system, including cubic and molecular-based equations of state such as van der Waals Eos,^31^ Peng–Robinson EoS,^32^ and the SAFT family EoS,^33−35^ just to mention a few examples. A key limitation of most EoSs is their inability to describe inhomogeneous systems, unless they are used along other methods such as classical density functional theory (DFT),^36−38^ which are numerically difficult to implement and restricted to simple geometries. However, by combining a SAFT EoS with the LDP-PF methodology for molecular simulation a model can be produced that simultaneously describes correct thermodynamics of the system while also allowing for the investigation of the density inhomogeneities often observed in soft matter formulations.^20,39^

In this work, the focus is to study the phase equilibria of water using the LDP-PF methodology coupled with the so-called SAFT-VR Mie EoS, which predicts the thermodynamic properties of complex fluids to high accuracy. A particular strength is its ability to describe association effects such as hydrogen bonding.^13,40−42^ The SAFT-VR Mie EoS,^40,43^ and its group contribution version known as the SAFT-γ Mie EoS,^41^ can accurately predict the bulk properties of a wide range of fluids, thus providing a robust underlying molecular model to the simulation method presented in this work. In this EoS, molecules are represented as chains of spherical segments that interact through the Mie potential^44^ to give a greater degree of accuracy compared to earlier versions of the theory using simpler potentials such as square well^45^ or hard-sphere potentials.^35^ The use of the Mie potential shows a significant improvement in the description of the second-derivative properties, such as isothermal compressibility, heat capacity, and speed of sound.^40,43^ The Mie potential, along with the SAFT-γ Mie EoS has also been successfully used to develop coarse-grained models to study complex molecular fluids.^10−13,15^ One would expect that the consideration of the association term in SAFT can yield an excellent EoS for the fluid phase of water.^42^ This would significantly improve previous simulation studies using pairwise potentials that do not explicitly consider hydrogen bonding, instead relying on effective interactions.^13^

The LDP-PF methodology is expected to give good equilibrium bulk properties according to the underlying EoS,^39^ however, this underlying EoS is often not parametrized to give good interfacial properties, such as surface tension. To remedy this, Warren^21^ used different sets of LDP parameters to adjust the interfacial properties. In this work, however, we correct the surface tension by considering a square gradient (SG) term.^46−50^ This approximation has been shown to suitably tune the surface tension to reproduce the interfacial properties of a real fluid. In particular, we apply the SG model of DeLyser et al.^47^

In this work, we apply the SAFT-VR Mie EoS as the underlying potential to the LDP-PF methology to accurately describe the thermodynamic properties of water in mesoscopic simulations and simultaneously access desirable time and length scales enabled by the DPD-style potentials. This model can be used as a foundation for more sophisticated simulations to describe complex formulations.

## 2 Methodology

### 2.1 The LDP-PF Potential

In this framework, molecules are represented as chains of spherical segments (sites). In the special case of water, a single-site model is used. For the LDP-PF methodology, it is necessary to define a local density for every site in the system. The local density of particle *i*, denoted as ρ̅_*i*_, is given by
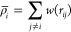
1where *r*_*ij*_ is the distance between particles *i* and *j* and *w* is a weighting function, chosen to be zero beyond a given cutoff distance *r*_c_, i.e., *w*(*r*_*ij*_) = 0 for *r*_*ij*_ > *r*_c_.^20,23^ For this work, following other works using a SG term,^46,48^ the Lucy weighting function is used,^51^ which is given by
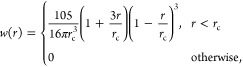
2where *w*(*r*) is also normalized, such that ∫*d***r***w*(*r*) = 1. Under the LDP-PF approximation, the potential energy *U*^LDP^ of the system is obtained as
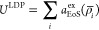
3Here *a*_EoS_^ex^(ρ) is the excess Helmholtz energy per particle, according to the EoS of choice, at a bulk density ρ. The basic idea of this approach is that if the interaction range, *r*_c_, is sufficiently large, then ρ̅ ≈ ρ, and the simulation reproduces the bulk thermophysical properties of the given, underlying EoS.^20,23^ In this particular case, the excess Helmholtz energy corresponds to the SAFT-VR Mie EoS.^40^

### 2.2 SAFT-VR Mie Parameters

In this work, three parametrizations of SAFT-VR Mie of water are considered, all based on single-site models (i.e., one water molecule per particle). Within the SAFT-VR Mie EoS, sites interact via the Mie potential ϕ^Mie^ given by
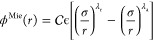
4where
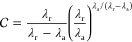
5ϵ and σ correspond to the depth and range of the potential, and λ_r_ and λ_a_ are the exponents controlling the repulsive and attractive contributions of the Mie potential, respectively. In the SAFT-VR Mie EoS, the excess Helmholtz free energy *a*_SAFT_^ex^ is given by^40,41^
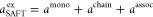
6where *a*^mono^ is the monomer contribution, denoting the excess free energy contribution per particle, of a fluid of Mie monomers, *a*^chain^ is the chain contribution from the formation of chains, and *a*^assoc^ is the associative contribution from hydrogen bonding. Since the models used in this work represent water as a single-site, *a*^chain^ = 0 in all cases.

The three SAFT-VR Mie CG water models for molecular simulation used in this work correspond to the two temperature-dependent models reported by Lobanova et al.^13^ and the Associating model reported by Dufal et al.^42^ The details of these three models are presented in the Appendix. In the following, these models are referred to as (a) the vapor–liquid equilibrium (VLE) model, (b) the interfacial tension (IFT) model, and (c) the Associating model, respectively. In both the VLE and IFT models, water is represented as a single-site particle interacting through the Mie potential ϕ^Mie^(λ_r_ = 8, λ_a_ = 6) and without considering the association term, i.e., *a*^assoc^ = 0. Since not a single set of values for the parameters ϵ and σ in eq 4 can represent the saturation properties of water over a wide range of conditions, Lobanova et al. reported two models with temperature-dependent parameters ϵ(*T*) and σ(*T*), where *T* is the absolute temperature. The first CG model (the VLE model) was fitted to represent vapor–liquid equilibria using liquid density ρ_liq_ and vapor pressure *p*_v_ with high accuracy, but exhibiting a poor representation of interfacial tension, while the second CG model (the IFT model) was fitted to represent saturation liquid density ρ_liq_ and interfacial tension γ with high accuracy, but the model showed a poor representation of the vapor pressure *p*_v_.

The third and final model considered in this work is the associating model of Dufal et al. that represents water as a single-site particle interacting via the Mie potential ϕ^Mie^(λ_r_ = 17.02, λ_a_ = 6) and also explicitly includes hydrogen bonds through the association term *a*^assoc^ in eq 6 using four square-well sites, two of which represent hydrogen atoms and the other two representing the two lone pairs. The advantage of this model over Lobanova’s models is that a single set of molecular parameters can be used over a wide range of conditions of temperature and density. However, incorporating the SAFT-like association interactions in standard molecular simulation is not trivial, but it is straightforward in the LDP-PF approximation since only the excess free energy of the equation of state is needed.

#### 2.2.1 The Square Gradient Term

As noted previously, most EoSs are only parametrized for bulk properties and cannot be expected to correctly represent the surface tension. To enforce a better description of surface tension γ, we make use of the square gradient (SG) theory.^48,52^ Within SG theory, an additional term is added to the potential that allows γ to be tuned by an additional parameter, denoted by κ. The final form of the potential is given by
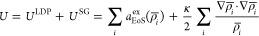
7where *U*^SG^ is the SG term and κ is a state-dependent coefficient. Ideally, *U*^SG^ should have little impact in regions where ρ is constant, i.e., in the bulk, but should only alter *U* in the interfacial region. This is indeed the case as discussed in detail in Section 3. The final surface tension γ obtained from simulations using the potential energy function *U* will therefore have two contributions: γ = γ_LDP_ + γ_SG_, where γ_LDP_ is the intrinsic surface tension obtained from the potential energy function *U*^LDP^, which becomes particularly large at low temperatures, and γ_SG_ is the correction term associated with the SG term *U*^SG^. The total surface tension γ of the resulting potential is found to increase approximately linearly with κ at a given temperature; therefore, several simulations are performed with varying values of κ to obtain the experimental surface tension of water (from NIST^53^) by linear interpolation. The choice of κ is found to reproduce the experimental data of water in only over a small temperature range. Therefore, the optimal values of κ have been determined for the temperature of 280–520 K, and a cubic fit of κ with *T* is used to give an estimate of κ(*T*) for a variety of *T*. The complete parametrization of κ for each model is presented in the Appendix. As shown previously,^39^ the calculated surface tension has a strong dependence on *r*_c_ (cf. eq 2). Thus, the parametrization for κ will also vary with *r*_c_.

### 2.3 Simulation Details

All simulations of the LDP-PF potential coupled with the SAFT-VR Mie EoS are performed using Monte Carlo (MC) simulations in the canonical *NVT* and isobaric–isothermal *NpT* ensembles, where *N* is the total number of particles in the system, *V* is the volume, *T* is the absolute temperature and *p* is the absolute pressure, using an in-house code. All simulations are performed using a cutoff of *r*_c_/σ(*T*) = 4.0 (cf. eq 2). This cutoff offers a compromise between achieving a good representation of bulk properties and not having a model that has excessively broad interfaces. To calculate the VLE properties of the systems, the direct coexistence method was used.^54^ In this method, a box of liquid is prepared, first placing the particles first in an orthorhombic box using PACKMOL^55^ and then a vacuum is added along the *z* direction such that the overall density of the system lies within the two-phase region. The final box has a volume *V* = *L*_*x*_*L*_*y*_*L*_*z*_, where *L*_*k*_ is the length of the simulation box along the *k* directions, and *L*_*x*_ = *L*_*y*_ < *L*_*z*_. This elongated system allows the formation of a planar interface between the coexisting vapor and liquid phases. The coexistence densities are obtained from the density profiles along the *z* direction calculated during the simulations. A total of *N* = 5000 particles has been used, with *L*_*x*_ = *L*_*y*_ = 3*r*_c_ and *L*_*z*_ adjusted to obtain an overall density value close to the critical density. The length of the simulation runs are given in MC cycles, where a cycle is defined as *N* attempts to displace a particle. Approximately 10^5^ MC cycles are used for equilibration of the system, and similar numbers for the production runs.

In order to calculate the surface tension γ, the test-area method is used.^56^ This method avoids the calculation of the virial tensor, and therefore the forces, by performing energy calculations of ghost deformations (perturbations) of the simulation box while keeping the total volume constant. This deformation can increase (+) or decrease (−) the interfacial surface area . Note that this deformation is not accepted during the simulation, and it is only used to evaluate the change of the potential energy during a deformation. For a planar interface with the normal direction along the *z* direction, the total interfacial area is , where the subindex 0 indicates the referenced (unperturbed) system. A change in the test area leads to a new area , where  is a dimensionless parameter that leads to a small fractional change in the area , while the dimension of the box in the direction *z* is adjusted to keep the volume constant. This deformation leads to , , and . Throughout this work, . The change in the Helmholtz free energy for such a perturbation, denoted by Δ*A*^±^, is
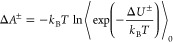
8where *k*_B_ is the Boltzmann constant, and Δ*U*^±^ is the change in potential energy of the system between the perturbed (1) state and the reference (0) state, i.e., Δ*U*^±^ = *U*_1_^±^ – *U*_0_. To improve the accuracy of the calculation, the surface tension is calculated through a central difference formula as
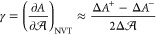
9

To calculate the vapor pressure *p*_v_ at a given temperature *T*, a box of vapor at the coexisting vapor density ρ_v_ is equilibrated, and then the pressure is calculated using the test volume method.^57^ The test volume method is similar to the test area method, except that the volume is no longer kept constant. The size of the volume perturbation is characterized by ζ = Δ*V*/*V* = Δ*L*_α_/*L*_α_, where Δ*V* is the difference in the volume of the perturbed state with respect to the reference state, *L*_α_ is the length of a box dimension along the direction α, and Δ*L*_α_ is the difference between the length of the perturbed box and the length of the reference box. In this case, ζ is taken to be positive. The *αα* component of the pressure tensor, *p*_*αα*_ is calculated using finite differences as
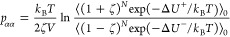
10where again Δ*U*^±^ is the change in potential energy between the perturbed and reference state. Note that in both the test area and test volume methods, the scaled position remains the same between the perturbed state and the reference state.

Finally, the isothermal compressibility β_*T*_, which along with the surface tension is a relevant property for the application of water models in soft matter, is calculated via simulations in the *NpT* ensemble using the following fluctuation relation^58−60^
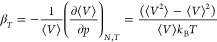
11

To quantify the performance of the prediction of our model of a given property *X*, we use the percent absolute average deviation (%AAD) defined as
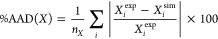
12where *X*_*i*_^exp^ represent the *i*-th experimental point, *X*_*i*_^sim^ is the *i*-th calculated point, and *n*_*X*_ is the number of data points.

## 3 Results and Discussion

### 3.1 Vapor–Liquid Equilibria

**Figure 1:**
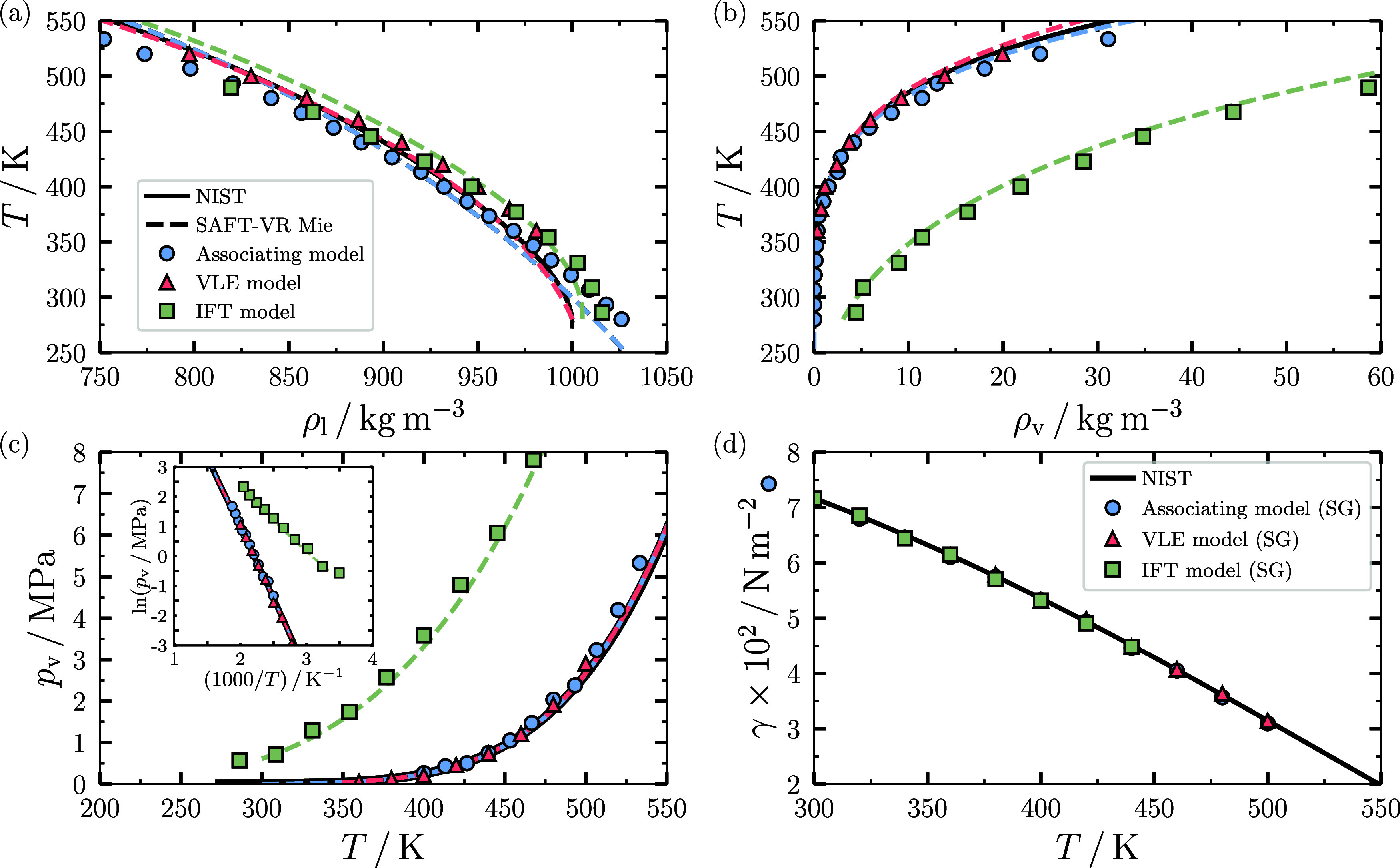
Results for the VLE of water. The results correspond to (a) liquid density ρ_l_, (b) vapor density ρ_v_, (c) vapor pressure *p*_v_, and (d) surface tension γ. In all plots, the symbols correspond to the MC simulations results obtained with the DLP-PF method using the SAFT-VR Mie EoS as the underlying potential: circles correspond to the Associating model of Dufal et al.,^42^ triangles and squares correspond to the VLE and IFT of Lobanova et al.,^13^ respectively. Uncertainties are proportional to the symbol size. The continuous black curve corresponds to experimental phase equilibria data from NIST,^53^ while the dashed curves in (a)–(c) correspond to the prediction of the SAFT-VR Mie EoS,^40^ using the same colors as their corresponding simulation model. The inset in (c) corresponds to the Clapeyron representation of the vapor pressure. In (d) the simulation results correspond to the same models as in (a)–(c) but adding the square-gradient term to the potential energy function (cf. eq 7).

In previous reports,^20,23,39^ it has been shown that the LDP-PF method, with a suitable choice of the cutoff radius *r*_c_ for the calculation of the local density, can reproduce bulk properties of an EoS of choice. It is expected that the molecular simulation methodology coupled with the SAFT-VR Mie EoS should yield similar thermodynamic results as the EoS, but with the added advantage of being able to describe inhomogeneous systems. This is particularly useful since implementing directly SAFT’s molecular model in standard computer simulations is not always straightforward. However, the LDP-PF methodology requires only the Helmholtz free energy of the EoS to represent the same system as the EoS in molecular simulation. The expectation is that, as all three SAFT-VR Mie models described in the Methodology have been shown to reproduce the properties of water (at least over the temperature range of the SAFT-VR Mie EoS parametrization and for those properties that have been fitted), the LDP-PF model should ideally reproduce the properties to a similar degree of accuracy to the EoS. It is natural, however, that the simulations will only ever be able to perform as well as the underlying EoS as this is the “ground truth” for the model. To demonstrate that this is indeed the situation, the case of water is showcased in this work due to the importance of developing accurate and efficient coarse-grained models of this molecule in soft matter. The results of the MC-*NVT* simulations, using the direct coexistence method for the LDP-PF using the Helmholtz free energy of the SAFT-VR Mie EoS as the underlying potential for water, are shown in Figure 1. The simulation results are for the three water models described in the Methodology, and these correspond to the Associating model of Dufal et al.,^42^ and the VLE and IFT models of Lobanova et al., respectively. The simulation results are compared with experimental vapor–liquid equilibria obtained from the NIST Webbook.^53,61^ As can be observed in Figure 1(a), the three models give an excellent prediction of the liquid ρ_l_ density, which is not surprising since the three models have been parametrized for this property. The three models exhibit %AAD values of less than 1.52% over a wide range of temperatures as reported in Table 1 for this property and with the larger deviations observed at high temperatures. For the vapor density ρ_v_ shown in Figure 1(b), both the Associating and VLE models show an excellent agreement when compared to experimental data, with an %AAD of 26.8% for the Associating model and 5.61% for the VLE model. However, the IFT model clearly exhibits a poor representation of the vapor density since this model has been fitted to represent only liquid density and surface tension.^13^ A similar observation can be drawn from the representation of the vapor pressure *p*_v_, shown in Figure 1(c), where it can observed that both the Associating model and the VLE model exhibit %ADD of less than 8.4%, while the IFT model is unable to represent this property to any reasonable degree of accuracy. Even in the Clapeyron representation, shown in the inset in Figure 1(c), one can observe that both Associating and VLE models exhibit a good representation of the vapor pressure at low temperatures, while the IFT model exhibits a poor prediction.

**Table 1:** MC-*NVT* Results for %ADD of the VLE Properties of Water Using the Associating, VLE, and IFT Models^a^

model	square gradient	%AAD ρ_l_	%AAD ρ_v_	%AAD *p*_v_	%AAD γ	*T*/K range
Associating	No	1.52	26.8	8.41		280.0–533.4
Associating	Yes	1.00	34.5	28.4	0.699	280.0–500.0
VLE	No	0.946	5.61	6.71		360.0–520.0
VLE	Yes	1.21	7.49	12.5	0.4603	360.0–500.0
IFT	No	1.40	6900	6598		286.3–489.6
IFT	Yes	3.03	9495	9390	0.4725	280.0–440.0

a The temperature range explored for each model is also indicated in the last column. The results for both the simulations without (eq 3) and with (eq 7) the square-gradient term are presented.

The three models exhibit large deviations from the experimental data as they approach higher temperatures. This is due to the inability of the underlying equations of state to accurately describe the critical region.

Despite the Associating models exhibiting slightly higher values of %AAD than the VLE model for the coexistence densities and the vapor pressure, the former model is preferable because (a) the range of temperatures accessible to this model is much broader, from 280 to 533 K (the other two models can only be used in the region where they have been parametrized), and (b) it does not require temperature-dependent Mie parameters, i.e., a single set of parameters in the associating models is enough to represent water over a wide range of conditions, as reported by Dufal et al., using the SAFT-VR Mie EoS representation for the same model. To demonstrate that the simulation results of the LDP-PF methodology can represent the same level quality of prediction as the SAFT-VR EoS, the results of the EoS for each model are also presented in Figures 1(a)-(c). It can be observed that the simulation results follow the predictions of the SAFT-VR Mie EoS very closely, with some deviations observed at very low or high temperatures. However, it is encouraging to see such a good agreement since it reflects that the correct representation of a system depends on the selected EoS.

As discussed in the Methodology, the intrinsic surface tension obtained from eq 3, i.e., the intrinsic surface tension γ_LDP_, does not represent correctly the surface tension of water and corrections through the SG term (cf. eq 7) are needed. The SG term used in this work could be considered a specific case of the general square gradient expression proposed by DeLyser et al.^47^ It is important to highlight that it was not possible to use a single value of the SG coefficient κ that can be used for all coexisting temperatures to represent the surface tensions satisfactorily. Therefore, the optimal value of κ at each temperature has been determined and subsequently, κ(*T*) is fitted to a polynomial, which is reported in the Appendix. This is somewhat in contrast to the theory, which predicts κ to be a temperature-independent function, at least to a simple approximation.^62^ However, this is likely not to be the case in the Associating model, due to the Arrhenius-like form of the association term. It is also important to note that the surface tension increases approximately linearly with κ at a given value of *r*_c_ and at a given temperature. This is very convenient from a practical point of view, as it allows for the easy determination of an appropriate parameter κ for a particular fluid. An open question is the nature of the individual contributions of *U*^SG^ and *U*^LDP^, to the surface tensions, as the total surface tension is made up of contributions from both. It is easy to imagine that a system where *U*^SG^ dominates over *U*^LDP^, or vice versa, may have different behavior in more complicated systems. This investigation will form part of a future publication in which more complicated systems will be considered.

The results for the surface tension γ predicted by the three models using the LDP-PF method plus the SG potential are presented in Figure 1(d). The results are compared to the experimental values obtained from NIST.^53^ The results for γ are very similar for the three models, which is expected as each model has been fitted with a temperature-dependent κ(*T*). The values of the state-dependent coefficient κ used in the SG term for each model are given in the Appendix. The %AAD for γ for each model are also presented in Table 1, where it can be observed that the three models perform similarly, with slightly better performance observed for the IFT model, which is the only of the three models parametrized for surface tension. However, the IFT model has been fitted to each temperature, so the fact that the Associating model exhibits a similar performance with a single parameter set is remarkable. Moreover, the use of a single parameter set is better from a pragmatic point of view, as the molecular parameters of the Mie potential do not change with temperature.

An aspect that is important to examine is the negligible effect the SG term should have on the bulk properties of water. Within the framework of a density functional theory, the density at a point corresponds to an ensemble average of a fluctuating density at that point. Thus, in a bulk phase, this density is constant, or, equivalently, ∇ρ (**r**) = 0. Thus, the SG term is zero except in the vicinity of an interface.^47^ In the simulated system, however, the local densities are fluctuating quantities and the SG term in the potential energy function will not average to zero. One would expect that these fluctuation effects would vanish in the bulk in the limit of large interaction distances. Our results show for *r*_c_ = 4 that such fluctuations have a negligible effect on the predictions of the bulk property. To this end, the calculations of the vapor–liquid equilibria using the direct coexistence method have been repeated using the SG term (cf. eq 7). The results for %AAD for the coexistence densities and vapor pressure of the three models are also reported in Table 1 where it can be observed that the prediction of properties remains very similar, although some small deviations are observed. The increase in %AAD for vapor density and vapor pressure, in particular, is due to the very small magnitude of these quantities at low temperatures.

### 3.2 Isothermal Compressibility

**Figure 2:**
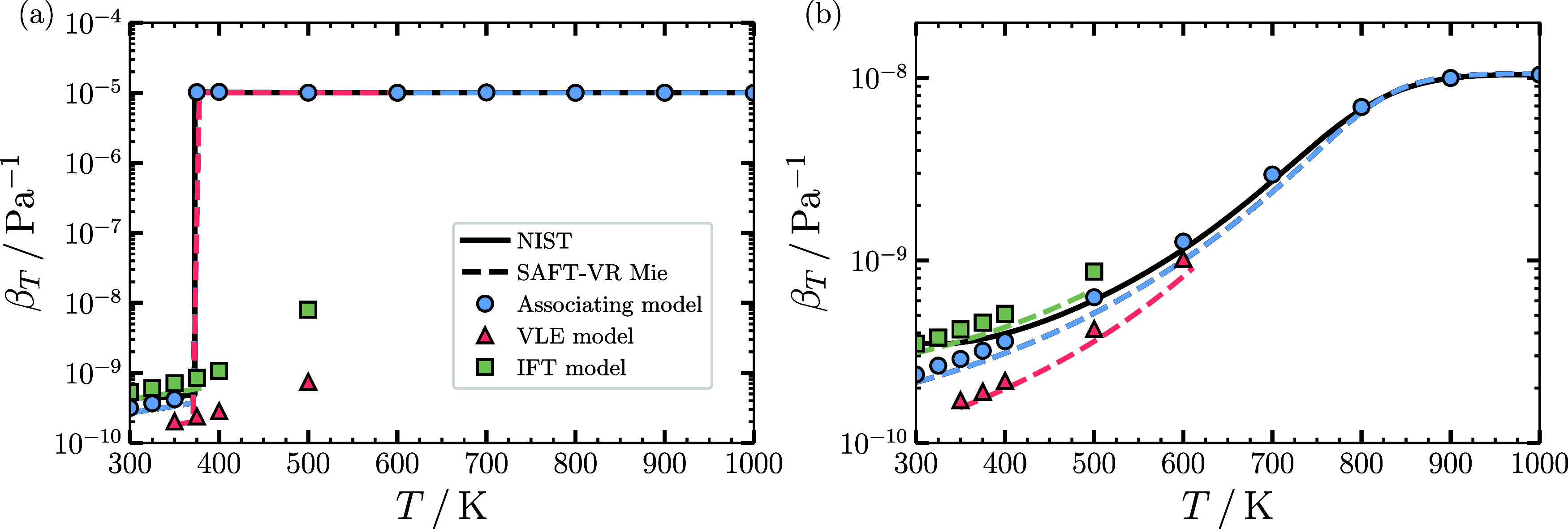
MC-*NpT* results for the isothermal compressibility β_*T*_ of water as a function of temperature *T* using the DLP-PF methodology using the SAFT-VR Mie EoS as the underlying potential. The results are for two isobars corresponding to (a) *p* = 0.1 MPa (subcritical isobar) and (b) *p* = 100 MPa (supercritical isobar). The circles correspond to the Associating model of Dufal et al., while the triangles and squares correspond to the VLE and IFT models of Lobanova et al.^13^ Uncertinties are proportional to the symbol size. In both figures, the continuous curve corresponds to experimental results obtained from NIST,^53^ while the dashed curves are the predictions using the SAFT-VR Mie EoS^40^ using the same colors as their corresponding simulation model.

Having demonstrated that the three models can reproduce the vapor–liquid equilibria of water in molecular simulation using the LDP-PF framework with similar accuracy as the underlying SAFT-VR Mie EoS, it is important to analyze how the models predict other thermodynamic properties of water, which have not been used for the parametrization of the models. Of practical importance for water models used in soft matter is the isothermal compressibility β_*T*_. Therefore, this property has been studied using the three water models via the LDP-PF framework. Simulations have been carried out in the *NpT* ensemble with temperature increments starting from approximately 300 K, and the isothermal compressibility obtained from density fluctuations via eq 11. The results are obtained for two isobars corresponding to *p* = 0.1 MPa (atmospheric pressure) and *p* = 100 MPa (chosen so the supercritical phase can be examined). The simulation results for both isobars are presented in Figure 2. As can be observed in Figure 2(a), the Associating model offers an excellent prediction of the isothermal compressibility of both vapor and liquid phases at atmospheric pressure when compared to experimental data from NIST^53^ over the entire range of temperatures corresponding to 300 to 1000 K, followed by the IFT model and the VLE models. For both IFT and VLE models, only the compressibility of the liquid phase is analyzed since the parametrization of the temperature-dependent Mie parameters of these models cannot be used beyond the temperature range used for their fitting. The results for %AAD of the three models for the liquid phase are presented in Table 2, where it can be observed that the Associating model offers the best prediction among the three models with %AAD = 18.21, followed by the IFT model with %AAD = 36.92 and the VLE model with %AAD = 55.79. It is also remarkable that the simulation results of the three models match the same predictions of the same models using the SAFT-VR Mie EoS, which are indicated by the dashed curves, indicating that the deviations from the experimental data are not due to the LDP-PF technique but rather to the fitting of the underlying water model.

**Table 2:** MC-*NpT* Results for % AAD of the Isothermal Compressibility β_*T*_ of Water Using the Associating, VLE, and IFT Models at a Subcritical (*p* = 0.1 MPa) and At A Supercritical (*p* = 100 MPa) Isobars^a^

		*p* = 0.1 MPa		*p* = 100 MPa	
model	square gradient	*T*/K	%AAD β_*T*_	*T*/K	%AAD β_*T*_
Associating	No	300–350	18.21	300–1000	11.33
Associating	Yes	300–350	28.50	300–1000	13.13
VLE	No	350–350	55.79	350–600	37.44
VLE	Yes	350–350	60.29	350–600	43.62
IFT	No	300–350	36.92	300–500	20.44
IFT	Yes	300–350	12.69	300–500	10.99

a The temperature range explored for each model is also indicated. For the subcritical isotherm, only the results for the isothermal compressibility of the liquid phase are presented. The results for both the simulations without (eq 3) and with (eq 7) the square-gradient term are presented.

A similar observation can be drawn from Figure 2(b) for the supercritical isobar at *p* = 100 MPa. Despite the high pressure, both the associating (%AAD = 11.33) and IFT (%AAD = 20.44) models offer a good representation of the isothermal compressibility, followed by the VLE model (%AAD = 37.44). However, here clearly the associating model is superior since it can be used even at extremely high temperatures compared to the VLE and IFT models, which can only be used within the temperature range for which they have been parametrized.

The effect of the SG term on the isothermal compressibility has also been assessed, and the results for %AAD are summarized in Table 2. At first glance, it could be assumed that the SG term affects the prediction of the isothermal compressibility, which is a bulk property. However, in reality, the prediction of the models is very good and the large deviation reflected in the values of %AAD is due to the extremely small magnitude of the isothermal compressibility observed in the liquid phase. Although only results for isothermal compressibility have been analyzed, in principle, other second derivative properties could be calculated, but this has been omitted as both the VLE and IFT models have a complex implicit dependence on *T* in the Mie parameters and even the associating model has dependencies on *T* in the evaluation of *a*_EoS_^ex^ from SAFT-VR Mie. Therefore, for properties such as residual heat capacity or thermal expansivity, which are calculated as fluctuations in thermal quantities, extra terms would enter the expressions for these quantities. This analysis goes beyond the scope of this work.

Before drawing conclusions from this work, it is necessary to discuss some theoretical and practical aspects of the three models considered in this work. The first aspect is the complex temperature dependence of the Mie parameters σ and ϵ required in both the VLE and IFT models. This dependency is straightforward to consider in MC-*NVT* simulations but requires careful consideration when using molecular dynamics (MD). It is for this reason that the Associating model is overall a better choice since the Mie parameters are constants. Second, from a practical point of view, the Associating model performed better or comparably over all the properties investigated in this paper and is not constrained by the temperature range over which the other models were fitted. Third, because of the ultrasoft nature of the potential, this model is ideal for simulations involving the insertion/deletion of particles to investigate the properties of molecular systems, such as in grand-canonical and Gibbs ensemble simulations. Finally, the calculation of the potential energy at every MC step, or at every time step in MD is a very expensive operation compared to simple pair potentials, but this issue can be overcome by using look-up tables for *a*_EoS_^ex^(ρ) to avoid performance penalty for the use of the more complicated model. For mixtures, where the composition of the species adds additional dimensions, the use of artificial neural networks trained with the SAFT-VR Mie free energy is an efficient option that can be considered.

## 4 Conclusions

In this work, the application of the LDP-PF framework coupled with the SAFT-VR Mie EoS has been applied to three water models reported in the literature, namely the Associating model of Dufal et al.,^42^ and the two temperature-dependent models of Lobanova et al., one parametrized for bulk VLE properties (VLE model) and the second for surface tension (IFT model). In general, the simulation results demonstrate that the three models offer an excellent representation of the saturated liquid density of water compared to the experimental data from NIST,^53^ but only the Associating and VLE models offer a good representation of the vapor density and vapor pressure of water, which is expected since they were parametrized for bulk VLE properties. The three models also offer good representations of the isothermal compressibility of water, with the Associating model exhibiting the best performance. Overall, it is recommended to use the Associating model as this only requires a unique set of Mie parameters, which is in contrast to the VLE and IFT models, which can only be used for temperatures within the range of the original parametrization. Even more importantly, the simulation results of all three water models are in very good agreement with the prediction of the bulk VLE properties obtained with the SAFT-VR Mie EoS, which demonstrates that the deviations observed between simulation results and experiments are mainly due to the representation of the free energy of the underlying model rather than the simulation methodology in itself. This opens an excellent methodology for developing coarse-grained models for mesoscopic simulations since any arbitrary EoS can be used to develop molecular or even empirical models, and the free energy of this model can be used in the LDP-PF framework to study inhomogeneous systems of the same models.

## Appendix. A Potential Parameters

In this section, the molecular parameters for the three water models are described. For the associating model, the SAFT parameters have been obtained from Dufal et al.,^42^ which correspond to σ = 3.0063 Å, ϵ/*k*_B_ = 266.68 K, and λ_r_ = 17.020 and λ_a_ = 6. The parameters for the association interaction are set to ε_HB_/*k*_B_ = 1985.4 K and *K*_HB_ = 101.69 Å^3^, where ε_HB_ and *K*_HB_ are the energy depth of the association potential and the volume of association bonds, respectively. The model has two hydrogen-bonding donors and two hydrogen-bonding acceptors.

The two temperature-dependent models of Lobanova et al.,^13^ i.e., the VLE and IFT models, use the same Mie exponents, namely λ_r_ = 8 and λ_a_ = 6. Neither model incorporates association terms in the Helmholtz energy expression, and therefore water molecules are modeled as simple spheres. To compensate for and improve the accuracy of the simple potential, each model has molecular parameters dependent on the temperature of the Mie potential. For the VLE model, these expressions are as follows:

13

which can only be used in the temperature range 343 ≤ *T*/K ≤ 613. For the IFT model, the molecular parameters are

14

which are applicable in the temperature range 293 ≤ *T*/K ≤ 493.

For the calculation of the surface tension, the coefficient κ in the square-gradient term has been parametrized at each temperature and is given by the following expression

15

where *c*_*i*_ are the fitted coefficients, which are reported in Table 3 for each model.

**Table 3:** Polynomial Coefficients of Each Model Used to Calculate the Square-gradient Coefficient κ as a Function of Temperature *T* for the Associating, VLE and IFT Models

model	*c*_3_	*c*_2_	*c*_1_	*c*_0_
Associating model	−1.1058 × 10^–7^	1.6504 × 10^–4^	–0.9515	25.7258
VLE model	–3.4716 × 10^–8^	7.6157 × 10^–5^	–0.0516	11.5662
IFT model	–1.1403 × 10^–7^	2.4507 × 10^–4^	–0.1515	37.9433
